# Editorial Notes

**Published:** 1882-04

**Authors:** 


### EDITORIAL NOTES.

A NEW museum is to be built for the University of Michigan at a cost of $60,000.

LAWSON TAIT speaks of Listerism as one of the largest, best blown and most attractive bubbles ever displayed to a surgical audience.

OF the fifty-six Professors of Harvard College, forty-three are graduates of Harvard—a notable instance of an Alma Mater appreciating her own children.

SMALL-POX has been effectually stamped out in this city, not a case now existing. The Health Physician, Dr. Phelps, recommends that the name of the Pest House be changed to Quarantine Hospital, a very good suggestion, which we hope will be adopted.

THE necrology of the past month includes the name of the eminent Dr. Pancoast, of Philadelphia, whose skill in the various departments of surgery has given him more than an American reputation. Among the readers of the JOURNAL are probably many who have listened with profit and pleasure to his clear demonstrations of technical subjects, and, having had personal knowledge of his ability, can measure the loss the profession has sustained by his death.

A LATE number of the *American Journal of Obstetrics* contains an exhaustive and valuable article by Dr. Garrigues, of New York, on the "diagnosis of ovarian cysts by means of the examination of the contents." His conclusion is that the examination of the fluid affords a very valuable aid to diagnosis, but that it would be rash to base a diagnosis on the character of the fluid alone. This does not differ much from the opinion of Baker Brown; that "microscopic examination may serve to strengthen an opinion—but alone it ought not to decide one."

"MEDICAL EDUCATION" seems to have received its full share of attention in Commencement addresses this year, and we are glad to see a general recognition of the fact that the granting of medical degrees by faculties, having a *financial interest* in the college and in the *number of graduates*, is one of the potent factors in maintaining the present condition of things. The appointment of a Board of Examiners, before whom all candidates for degrees would have to appear, would crowd out a good many colleges from the doctor-making business.

DR. PARVIN, (ex-President American Medical Association), says that the New York State Medical Society, in voting to consult with homœopaths, has placed itself outside of the American Medical Association, and that they must be glad to rescind their action before two years, for the great mass of practitioners in New York State were loyal to the old flag, though a few specialists, hungry for homœopathic consultations, may dishonor or desert. Homœopathy deserves no such concession as the New York gentlemen wish to make. Honestly practiced it is absurd, dishonestly it is knavery, at best it has been a foolish fashion and is a dying faith.

THE effort in the legislature to annul the registration of physicians, as required by the medical law of 1880, was considered at a recent meeting of the Erie County Medical Society, and the following resolutions adopted:

*Whereas,* A bill now pending in the Legislature to regulate the practice of medicine and surgery in this State gives the right to every person to obtain medical advice and aid from any one in whom he may repose confidence whether he is registered or not as a physician, thereby removing all restrictions in regard to the practice of medicine and annulling the registry law passed last year by which some protection was secured to the public at large against the dangerous practices of ignorant and unprincipled persons; therefore,

*Resolved,* That the Society request our Senator and Members of Assembly from this county to use all honorable means to defeat the passage of said bill, and that the Secretary forward this resolution to said members

A Chicago paper reports the death by small-pox in that city of a physician quite prominently known for his anti-vaccination sentiments. It would be well if retribution followed every fault of judgment as closely as in this case, since the superstition of some ignorant practitioners has immolated many victims on the altar of this loathsome disease and been an important factor in producing its epidemic form. No less excellent would be an enactment such as our Health Physician has recommended, making the refusal to submit to vaccination a misdemeanor in the eyes of the law.

